# Efficacy and safety of different antithrombotic regimens including direct oral anticoagulants for the prevention of portal vein system thrombosis after splenectomy in patients with liver cirrhosis and portal hypertension: a systematic review and network meta-analysis

**DOI:** 10.1097/JS9.0000000000003220

**Published:** 2025-09-18

**Authors:** Lu Cao, Kun Wei, Jun Hai, Zhaoqing Du, Nan Zhou, Peng Zhang, Dan Zhang, Yu Zhang

**Affiliations:** a Department of Pharmacy, Shaanxi Provincial People’s Hospital, Xi’an, Shaanxi Province, China; b Department of Hepatobiliary Surgery, Shaanxi Provincial People’s Hospital, Xi’an, Shaanxi Province, China

**Keywords:** cirrhosis with portal hypertension, DOACs, network meta-analysis, PVST, splenectomy

## Abstract

**Objective:**

To systematically evaluate the efficacy and safety of different antithrombotic regimens for preventing portal vein system thrombosis (PVST) formation in patients with cirrhosis and portal hypertension undergoing splenectomy using a network meta-analysis method based on published literature.

**Methods:**

A comprehensive search was conducted using PubMed, Web of Science, EMBASE, Cochrane Library, and three Chinese databases (CBM, CNKI, and Wanfang Data) from inception to June 2025. Studies involving different prophylactic antithrombotic regimens for patients with cirrhosis and portal hypertension who underwent splenectomy were included. Literature screening and assessment of the methodological quality of the included studies were performed using the Cochrane systematic review method, and characteristic information was extracted for network meta-analysis. The clinical outcomes included the incidence of PVST at postoperative month 1 (POM 1), postoperative month 3 (POM 3), and postoperative month 6 (POM 6), the incidence rate of bleeding and the course of antithrombotic prophylaxis.

**Results:**

A total of 19 studies involving 1968 patients were included. Nine different prophylactic antithrombotic regimens were analyzed, including antiplatelet therapy (APT) alone; anticoagulation alone, including low-molecular-weight heparin (LMWH) + warfarin, LMWH + rivaroxaban, and rivaroxaban alone; anticoagulation combined with APT, including LMWH + APT, LMWH + warfarin + APT, LMWH + apixaban + APT, and rivaroxaban + APT; and placebo. The results of the network meta-analysis and direct meta-analysis revealed that rivaroxaban + APT had the best efficacy in reducing PVST-POM 1, PVST-POM 3, and PVST-POM 6, followed by LMWH + warfarin + APT. The efficacy of APT alone and placebo was relatively poor. The incidence rate of bleeding was similar among the different prophylactic antithrombotic regimens. Three months of postoperative prophylactic antithrombotic therapy could significantly reduce the incidence of PVST, whereas extending the course to 6 months provided no further reduction of the incidence of PVST.

**Conclusion:**

For patients with cirrhosis and portal hypertension undergoing splenectomy and with Child‒Pugh class A–B liver function, prophylactic antithrombotic regimens combining anticoagulation and APT, especially rivaroxaban + APT, show the best efficacy, significantly reducing the incidence of PVST without increasing the risk of bleeding. A 3-month postoperative prophylactic antithrombotic therapy may be the optimal preventive course.

## Introduction

Splenectomy is a common surgical treatment for liver cirrhosis complicated by portal hypertension, splenomegaly, splenic rupture, and other related conditions. Portal vein system thrombosis (PVST) is a common postoperative complication, and prospective cohort studies show an incidence ranging from approximately 1.6% to 24.4%^[1–3]^. PVST increases the risk of ischemic bowel necrosis, esophageal variceal rupture, decompensation of cirrhosis, and even death^[4]^, so timely and effective prevention and treatment are particularly important.

Several meta-analyses have identified risk factors for PVST following splenectomy, including increased portal vein diameter, decreased portal vein blood flow velocity, thrombocytopenia, and splenic enlargement or intimal hyperplasia^[5–7]^. The Virchow theory suggests that venous thrombosis is associated with hemodynamic changes, endothelial injury, and a hypercoagulable state. Intrahepatic fibrosis, destruction of hepatic sinusoids, and vascular torsion and occlusion are common among patients with cirrhosis, and Doppler ultrasound showing a portal vein blood flow velocity less than 15 cm/s is considered a critical threshold for predicting PVST, with the associated risk increasing 10–20-fold^[8,9]^. Portal hypertension leads to a decrease in blood flow velocity and an increase in vascular shear stress. Endoscopic, interventional, and surgical procedures can cause local endothelial injury in blood vessels, triggering the release of coagulation factors and the initiation of thrombosis. The risk of PVST is more than 10 times greater following either open or laparoscopic splenectomy^[10]^. In cirrhotic patients, the synthesis of coagulation factors, anticoagulants, and fibrinolytic substances decreases in parallel, resulting in a “temporarily fragile” rebalanced coagulation state. This delicate balance is easily disrupted, leading to the simultaneous presence of both hypercoagulable and hypocoagulable states. Moreover, elevated levels of gut-derived endotoxins in cirrhotic patients induce thrombin generation and increase the levels of von Willebrand factor (vWF)^[11]^, and mutations in coagulation factors further contribute to a hypercoagulable state.

Pathophysiological studies have suggested that PVST differs from classical venous thromboembolism (VTE) in certain aspects. The portal vein, which is approximately 6–8 cm in length and approximately 1 cm in diameter, together with the hepatic artery, drains blood into the hepatic sinusoids of the liver lobules. Unlike other deep veins, the portal vein lacks valves that prevent blood reflux or promote blood return to the heart. Instead, it generates pressure through the inflow of blood and outflow resistance. In cirrhosis, fibrous bands and regenerating hepatic cell nodules compress the hepatic sinusoids, causing narrowing or occlusion and obstructing portal vein blood flow, resulting in increased portal pressure. The development of arteriovenous shunts between the hepatic artery and small portal vein branches further elevates portal pressure as high-pressure hepatic arterial blood flows into the low-pressure portal veins. When the venous system can no longer withstand this high pressure and load, compensatory changes, including venous wall dilation, remodeling, and structural changes, along with the extravasation of red and white blood cells and collagen deposition, occur. Some researchers have argued that the proliferation of the portal vein intima and portal hypertension may be more important factors contributing to the development of PVST.

Understanding the components of PVST thrombi is crucial for prevention and treatment. Driever’s study^[12]^ demonstrated that approximately one-third of PVST patients have fibrin-rich thrombi at the top of the fibrous intima. In some cases, these fibrin-rich regions also contain vWF and CD61, which mediate platelet aggregation and adhesion. Interestingly, polyhedral red blood cells, which are markers of arteriovenous thrombosis contraction, are rarely observed^[13]^. These characteristics suggest that PVST may differ from deep vein thrombosis, potentially requiring different treatment strategies. There are no existing standardized pharmacological regimens for the prevention of PVST following splenectomy. Clinically reported strategies include the use of aspirin, dipyridamole, low-molecular-weight heparin (LMWH), warfarin, rivaroxaban, and various combinations of these drugs with different dosages and treatment durations. However, the effectiveness of these treatment protocols in preventing PVST and the safety concerning bleeding risks remain unclear. We conducted a network meta-analysis of these issues to provide evidence for the postoperative prevention of PVST in patients who underwent splenectomy for liver cirrhosis and portal hypertension. The work has been reported in line with the TITAN criteria^[14]^.

**HIGHLIGHTS**

- Our findings indicate that for patients with cirrhosis and portal hypertension undergoing splenectomy, prophylactic antithrombotic regimens combining anticoagulation and APT, especially rivaroxaban + APT, show the best efficacy, significantly reducing the incidence of PVST and improving the occurrence of cirrhosis decompensation, without increasing the risk of bleeding.
- Our findings indicate that the prophylactic antithrombotic regimens using APT alone are similar to placebo, both showing poor efficacy and significantly increasing the incidence of PVST in these patients.
- For patients with liver cirrhosis and portal hypertension who undergo splenectomy, prophylactic antithrombotic therapy for 3 months after surgery can significantly reduce the incidence of PVST, while extending the duration of prophylactic antithrombotic therapy to 6 months does not reduce the incidence of PVST.
- This study provides strong evidence for the prevention of PVST after splenectomy for liver cirrhosis, and offers the optimal antithrombotic regimen for preventing PVST for patients in this category after surgery.

## Materials and methods

Our study has been reported in line with the STROCSS guidelines^[15]^. This study was registered on the PROSPERO database and reported in line with the PRISMA (Preferred Reporting Items for Systematic reviews and Meta-Analyses)^[16]^ and AMSTAR (A Measurement Tool to Assess Systematic Reviews) guidelines^[17]^.

### Inclusion criteria

(1) Participants: Patients aged ≥18 years, diagnosed with liver cirrhosis complicated by portal hypertension through clinical evidence, imaging findings, and histological examination; with Child‒Pugh class A–B liver function, secondary hypersplenism and a platelet count ≤50 × 10^9^/L; a history of esophageal-gastric variceal bleeding; no PVST detected by imaging studies; and scheduled for elective splenectomy or splenectomy combined with periesophageal devascularization, pericardial devascularization, portal‒azygous devascularization, or other combined devascularization procedures^[18–22]^. There were no restrictions regarding sex or ethnicity.

(2) Interventions and comparisons: Postsplenectomy thromboprophylaxis, defined as the use of antithrombotic drugs before the formation of a PVST to prevent its occurrence. The different prophylactic thromboprophylaxis regimens defined in this study include the following nine commonly used strategies, especially direct oral anticoagulants (DOACs): antiplatelet therapy (APT) alone; anticoagulation therapy alone, including LMWH + warfarin, LMWH + rivaroxaban, or rivaroxaban alone; and the combination of anticoagulation and APT, including LMWH + APT, LMWH + warfarin + APT, LMWH + apixaban + APT, and rivaroxaban + APT. Antiplatelet drugs include aspirin, clopidogrel, or dipyridamole. The experimental group received one of the above regimens, whereas the control group received another regimen from the list or a placebo. The dose and duration of the interventions were not restricted.

(3) Outcomes: Primary outcomes: The incidence rates of PVST at postoperative month 1 (POM 1), postoperative month 3 (POM 3), and postoperative month 6 (POM 6) after splenectomy. Secondary outcomes: Incidence of bleeding during the treatment period and course of antithrombotic prophylaxis

(4) Study types: Published randomized controlled trials (RCTs) or retrospective cohort/case–control studies.

### Exclusion criteria

(1) Studies involving patients with liver malignancies or noncirrhotic conditions requiring splenectomy, such as trauma or malaria; (2) studies involving patients with preoperative confirmation of PVST or other deep vein thrombosis through Doppler ultrasound or abdominal CT scans; (3) studies where the thromboprophylaxis regimens included traditional Chinese medicine or proprietary Chinese medicine or where the description of the thromboprophylaxis protocol was unclear; (4) studies where the clinical outcomes did not align with the outcome measures defined in this study; (5) duplicate publications; (6) case report studies; (7) conference abstracts or study protocols; (8) studies for which the full text could not be obtained, even after contacting the authors; and (9) non-Chinese or non-English publications.

### Literature search strategy

The following databases were searched: PubMed, Web of Science, EMBASE, the Cochrane Library, and three Chinese databases (CBM, CNKI, and Wanfang Data). The search covered the period from inception until 19 June 2025. The search terms included (“splenectomy*”) AND (“portal vein thrombosis” OR “portal vein system thrombosis” OR “PVT” OR “PVST”) AND (“anticoagulant*” OR “anticoagulation*”). A combination of MeSH terms and free-text words was used, and the search was adjusted according to each specific database (please refer to the search terms in the Supplementary Digital Content Materials, available at: http://links.lww.com/JS9/F135). Additionally, the reference lists of eligible studies were reviewed to supplement the search.

### Study selection and data extraction

Two researchers independently screened the literature and extracted data, which were cross-verified. Any disagreements were resolved by consultation with a third researcher or by a fourth reviewer if necessary. Data extraction included the following: (1) Basic information: first author, publication year, and study design; (2) Clinical characteristics of the participants: age, specific surgical method, sample size, and disease type; (3) Interventions and comparisons: specific drugs, doses, and treatment duration; (4) Postoperative follow-up duration; and (5) Outcomes defined in this study.

### Methodological quality assessment of the included studies

The methodological quality of the RCTs was assessed using the Cochrane Risk of Bias 2.0 tool (RoB 2.0), which evaluates five domains: bias due to randomization, bias due to deviations from the intended interventions, bias due to missing outcome data, bias in the measurement of outcomes, and bias due to selective reporting^[23]^. For retrospective studies, the Newcastle‒Ottawa Scale (NOS) was used to evaluate the representativeness of the study population, comparability of the study groups, adequacy of follow-up duration, completeness of follow-up, and absence of attrition or withdrawal. A score of 5–9 points indicated a low risk of bias^[24]^.

### Statistical analysis

RevMan 5.3.3 and Stata 16.0 software were used in this study to perform direct and network meta-analyses.

First, for each outcome, studies comparing two antithrombotic regimens directly (with ≥2 studies) were included in a direct meta-analysis using RevMan 5.3.3 to compare efficacy and safety. All outcomes defined in this study were binary variables; thus, odds ratios (ORs) with 95% confidence intervals (CIs) were used to pool the data. The Cochrane *Q* test was used to assess heterogeneity among the included studies: when *P* > 0.1 and *I*^2^ ≤ 50%, no significant heterogeneity was present, and a fixed-effects model was used for the analysis; otherwise, a random-effects model was employed. A *P-*value < 0.05 indicated statistical significance.

Second, network meta-analysis using the “network” and “mvmeta” commands in Stata 16.0 was performed under the frequentist framework. This method employs restricted maximum likelihood to fit a multivariate random-effects meta-analysis model. A network plot was created to provide a simple overview showing all available evidence for the various interventions, with the size of the nodes representing the sample size and the thickness of the lines indicating the number of studies making direct comparisons. Since all outcome measures were binary variables, relative risks (RRs) with 95% CIs were used for pooling. For closed-loop outcomes, the node-splitting method was used to test for inconsistency, and consistency models were applied when *P* > 0.05. The results of two-by-two comparisons between different interventions are displayed in league tables. A surface under the cumulative ranking curve (SUCRA) was used to rank the interventions, with higher SUCRA values indicating a greater likelihood of a particular treatment being the most effective. Publication bias and small-sample effects were assessed using comparison-adjusted funnel plots, and the symmetry of the scatter distribution was qualitatively judged. Moreover, we combined the results of Egger’s and Begg’s tests performed using the metabias() function in R software version 4.4.2 to detect potential publication bias in the studies included in the network meta-analysis.

## Results

### Literature search results

**Figure 1.**
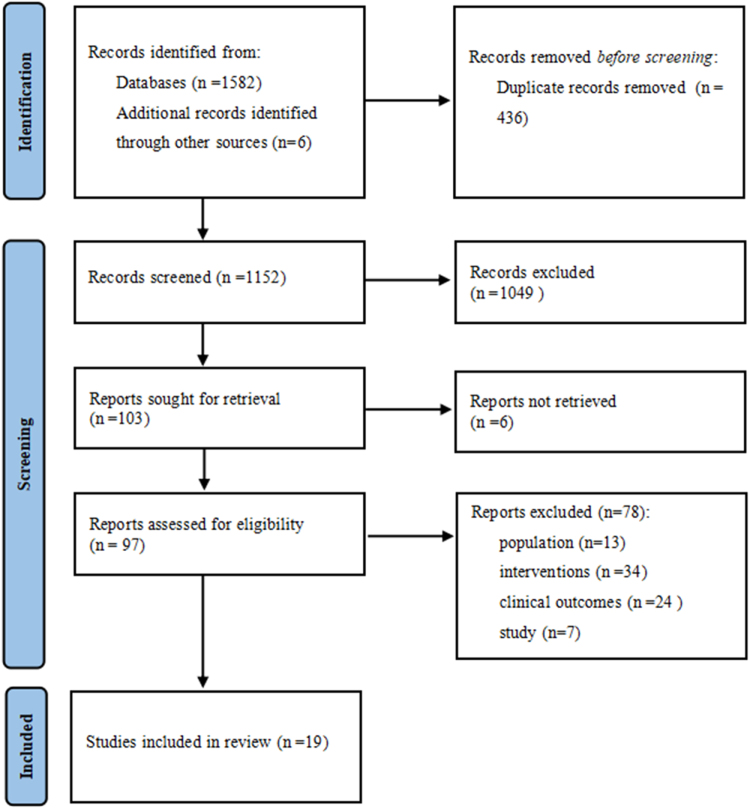
Flowchart of the literature selection.

The planned search terms were applied to the databases, and the references of the included studies were also reviewed. A total of 1588 articles were retrieved, including 796 in English and 786 in Chinese. After removing duplicate articles using EndNote X9, 1152 articles remained. The remaining articles were then screened according to the inclusion and exclusion criteria defined in this study. Finally, 19 studies^[18–22,25–38]^ were included in the network meta-analysis. The flowchart of the literature selection process is shown in Figure 1.

### Basic characteristics of the included studies and risk of bias assessment results

A total of 1968 patients were included in the 19 studies, with 1869 patients in the experimental groups, including 567 patients in the LMWH + APT group, 378 patients in the LMWH + warfarin + APT group, 356 patients in the APT group, 186 patients in the LMWH + rivaroxaban group, 174 patients in the rivaroxaban group, 114 patients in the rivaroxaban + APT group, 56 patients in the LMWH + warfarin group, 38 patients in the LMWH + apixaban + APT group, and 99 patients in the placebo group. In the study design, both the experimental and control groups considered patient demographics and disease types, ensuring comparability between the two groups at baseline. The basic characteristics of the included studies are shown in Table 1.

**Table 1:** Basic characteristics of the included studies

Author	Year	Study design	Age	Study population	Surgery type	Antithrombotic regimen		Treatment course	Follow-up duration	Outcomes	NOS score
						Experimental group	Control group				
Shi ZB	2024	Single-center RCT	55.74 ± 11.16 years	Cirrhosis with portal hypertension	Laparoscopic splenectomy	LMWH + apixaban + APT (*n* = 38)	LMWH + APT (*n* = 38)	3 months	6 months	PVST-POM 1	–
Feng YA	2024	Single-center RCT	43–54 years	Cirrhosis with portal hypertension	Splenectomy	Rivaroxaban (*n* = 60)	APT (*n* = 60)	6 months	6 months	PVST-POM 1/6, the incidence of bleeding	–
Wang D	2023	Single-center retrospective cohort study	46.42 ± 10.72 years	Cirrhosis with portal hypertension	Laparoscopic splenectomy and esophagogastric devascularization	LMWH + rivaroxaban (*n* = 186)	Rivaroxaban (*n* = 114)	3 months	10 years	PVST-POM 1	8
Lv Y	2023	Single-center RCT	18–80 years	Cirrhosis with secondary portal hypertension and splenomegaly	Splenectomy and perigastric devascularization	Rivaroxaban + APT (*n* = 41)	LMWH + warfarin + APT (*n* = 41)	1 month	6 months	PVST-POM 1/6	–
Li YN	2022	Single-center RCT	18–80 years	Cirrhosis with portal hypertension and splenomegaly	Laparoscopic splenectomy and pericardial devascularization	LMWH + warfarin + APT (*n* = 68)	LMWH + APT (*n* = 63)	6 months	18 months	PVST-POM 1/3/6	–
Zhen Y	2022	Single-center retrospective cohort study	28–64 years	Cirrhosis with hypersplenism	Splenectomy	LMWH + warfarin (*n* = 56)	Placebo (*n* = 50)	6 months	1 year	PVST-POM 1/3/6, the incidence of bleeding	9
Yao W	2021	Single-center RCT	18–80 years	Cirrhosis with portal hypertension	Open splenectomy and perigastric devascularization	Rivaroxaban + APT (*n* = 35)	LMWH + warfarin + APT (*n* = 35)	1 month	1 year	PVST-POM 1/3/6	–
Yao W-2	2021	Single-center RCT	18–80 years	Cirrhosis with secondary portal hypertension and hypersplenism	Open splenectomy and perigastric devascularization	Rivaroxaban + APT (*n* = 38)	LMWH + warfarin + APT (*n* = 38)	1 month	1 year	PVST-POM 1/3/6	–
Bai DS	2019	Single-center RCT	18–75 years	Cirrhosis with hypersplenism and platelet count < 50	Laparoscopic splenectomy and azygoportal disconnection	LMWH + warfarin + APT (*n* = 39)	LMWH + APT (*n* = 39)	12 months	2 years	PVST-POM 1/3/6	–
Chen YD	2019	Single-center retrospective cohort study	24–74 years	Cirrhosis with portal hypertension	Splenectomy and esophagogastric devascularization or combined disconnection	LMWH + APT (*n* = 31)	APT (*n* = 30)	3 months	3 months	PVST-POM 1/3	7
Xu TL	2017	Single-center retrospective cohort study	43.6 ± 8.5 years	Cirrhosis with portal hypertension	Splenectomy and disconnection	LMWH + APT (*n* = 42)	APT (*n* = 42)	6 months	6 months	PVST-POM 6	6
Jiang GQ	2016	Single-center retrospective cohort study	18–75 years	Cirrhosis with EGVB and secondary hypersplenism	Laparoscopic splenectomy and azygoportal disconnection	LMWH + warfarin + APT (*n* = 34)	LMWH + APT (*n* = 39)	12 months	1 year	PVST-POM 1/3	8
Jiang GQ-2	2016	Single-center retrospective cohort study	18–75 years	Cirrhosis with secondary hypersplenism	Laparoscopic splenectomy and azygoportal disconnection	LMWH + warfarin + APT (*n* = 35)	LMWH + APT (*n* = 40)	12 months	3 months	PVST-POM 1/3	8
Wu SL	2015	Single-center retrospective cohort study	27–63 years	Cirrhosis with portal hypertension	Splenectomy combined with pericardial devascularization	LMWH + Warfarin + APT (*n* = 52)	Placebo (*n* = 19)	1 month	2 years	PVST-POM 1	8
Chen HW	2015	Single-center retrospective cohort study	48.3 ± 12.4 years	Cirrhosis with portal hypertension	Splenectomy	LMWH + APT (*n* = 90)	APT (*n* = 46)	3 months	6 months	PVST-POM 1, the incidence of bleeding	7
Cheng Z	2015	Single-center retrospective cohort study	47.1 ± 13.3 years	Cirrhosis with portal hypertension	Splenectomy and esophagogastric disconnection	LMWH + APT (*n* = 139)	APT (*n* = 80)	1 month	1 month	PVST-POM 1, the incidence of bleeding	9
Liu XY	2013	Single-center RCT	≥18 years	Cirrhosis with portal hypertension	Splenectomy and perigastric devascularization	APT (*n* = 34)	Placebo (*n* = 30)	3 months	6 months	PVST-POM 3/6, the incidence of bleeding	–
Zou WX	2013	Single-center RCT	44.03 ± 9.65 years	Cirrhosis with portal hypertension	Splenectomy (or) plus azygoportal disconnection	LMWH + warfarin + APT (*n* = 36)	APT (*n* = 20)	3 months	3 months	PVST-POM 3	–
Li Y	2010	Single-center retrospective cohort study	40 ± 27 years	Cirrhosis with portal hypertension	Splenectomy and esophagogastric devascularization	LMWH + APT (*n* = 46)	APT (*n* = 44)	NA	6 months	PVST-POM 6, the incidence of bleeding	8

EGVB, esophageal variceal bleeding; NOS scores, Newcastle–Ottawa Scale scores, and scores of 5–9 indicating low risk of bias

**Figure 2.**
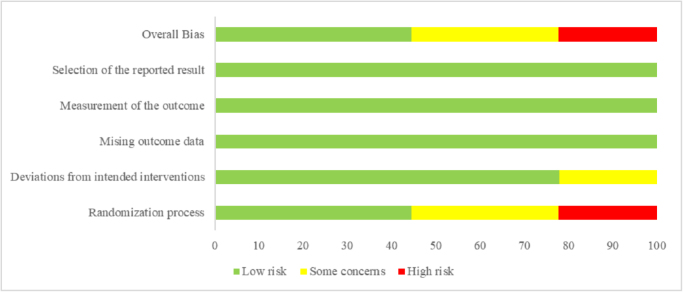
Bias analysis proportions of the included studies.

Among the 19 studies included, 9^[18,21,22,26,28,29,31,33,36]^ were RCTs. Of these, four studies^[18,29,31,36]^ were classified as low risk, three studies^[26,28,33]^ had some concerns, and two studies^[21,22]^ were classified as high risk. The specific risk of bias assessment results are shown in Figure 2. The remaining 10 studies^[19,20,25,27,30,32,34,35,37,38]^ were retrospective studies, with NOS scores ranging from 6 to 9, as detailed in Table 1. Overall, the methodological quality of the studies was acceptable.

### Meta-analysis

#### PVST-POM 1

##### Direct meta-analysis

**Figure 3.**
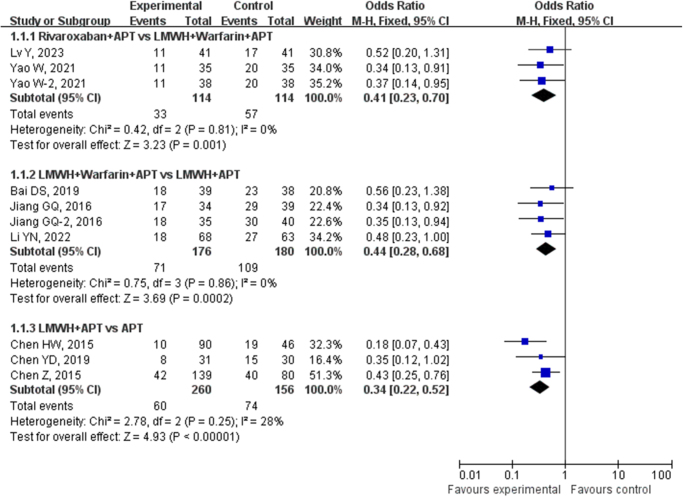
Direct meta-analysis comparison of different antithrombotic regimens in preventing PVST-POM 1.

A total of 3 studies involving 228 patients with cirrhosis and portal hypertension who underwent splenectomy were included in the current analysis. These studies compared the incidence rates of PVST-POM 1 between the use of rivaroxaban + APT and LMWH + warfarin + APT for prevention. No statistical heterogeneity was found among the included studies (*P* = 0.81, *I*^2^ = 0%), so a fixed-effects model was used for the analysis. The results of the direct meta-analysis indicated that the incidence of PVST-POM 1 in the rivaroxaban + APT group (28.95%) was significantly lower than that in the LMWH + warfarin + APT group (50.00%), and the difference between the two groups was statistically significant (OR: 0.41, 95% CI: 0.23–0.70, *P* = 0.001), as shown in Figure 3. A total of 4 studies involving 356 patients with cirrhosis and portal hypertension who underwent splenectomy were included in the current analysis. These studies compared the occurrence of PVST-POM 1 between LMWH + APT and LMWH + warfarin + APT for prevention. No statistical heterogeneity was found among the included studies (*P* = 0.86, *I*^2^ = 0%), so a fixed-effects model was used for the analysis. The direct meta-analysis results indicated that the incidence of PVST-POM-1 in the LMWH + warfarin + APT group (40.34%) was significantly lower than that in the LMWH + APT group (60.56%), and the difference between the two groups was statistically significant (OR: 0.44, 95% CI: 0.28–0.68, *P* = 0.0002), as shown in Figure 3. A total of 3 studies involving 416 patients with cirrhosis and portal hypertension who underwent splenectomy were included in the present analysis. These studies compared the occurrence of PVST-POM 1 between LMWH + APT and APT for prevention. No statistical heterogeneity was found among the included studies (*P* = 0.25, *I*^2^ = 28%), so a fixed-effects model was used for the analysis. The direct meta-analysis results indicated that the incidence of PVST-POM-1 in the LMWH + APT group (23.08%) was significantly lower than that in the APT group (47.44%), and the difference between the two groups was statistically significant (OR: 0.34, 95% CI: 0.22–0.52, *P <* 0.00001), as shown in Figure 3.

Sensitivity analysis was performed on the incidence of PVST-POM 1 in the rivaroxaban + APT group vs the LMWH + warfarin + APT group (Supplementary Digital Content Figure S1, available at: http://links.lww.com/JS9/F135), the LMWH + warfarin + APT group vs the LMWH + APT group (Supplementary Digital Content Figure S2, available at: http://links.lww.com/JS9/F135), and the LMWH + APT group vs the APT group (Supplementary Digital Content Figure S3, available at: http://links.lww.com/JS9/F135), and the results revealed no substantial changes in the pooled effect estimate, suggesting that the meta-analysis results were reliable.

##### Network meta-analysis

**Figure 4.**
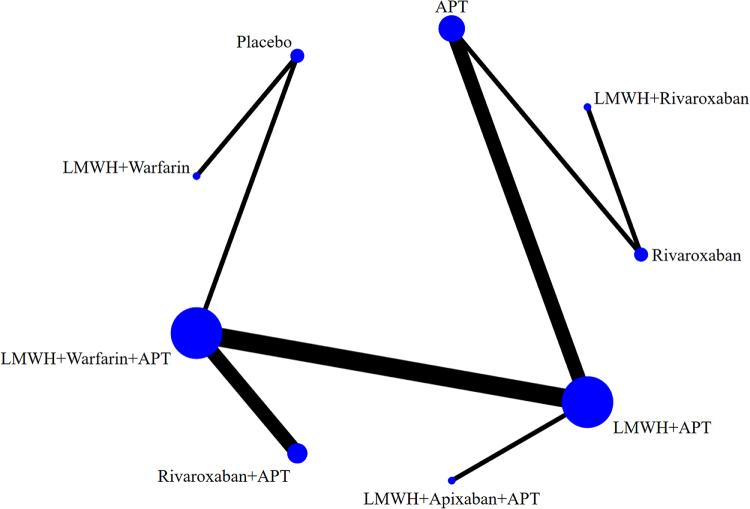
Network diagram of the occurrence of PVST-POM 1 under different antithrombotic regimens.

**Figure 5.**
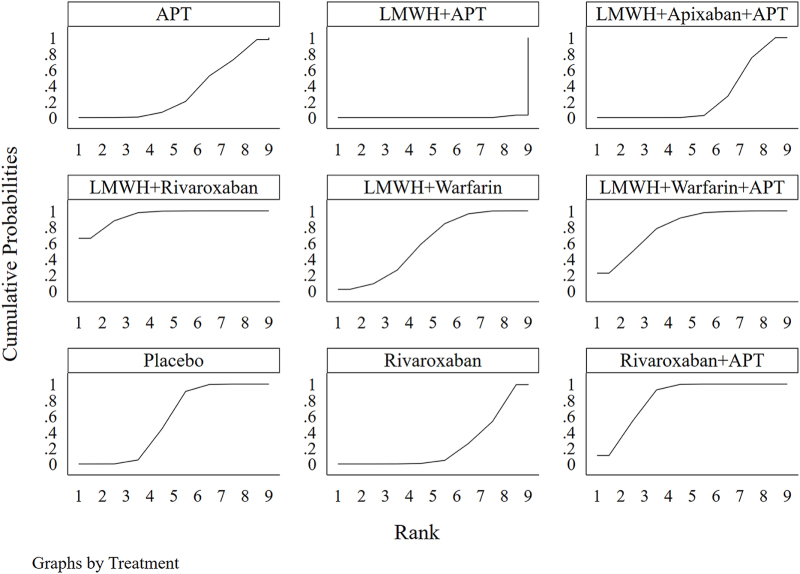
SUCRA of the occurrence of PVST-POM 1 under different antithrombotic regimens.

The network evidence diagram (Fig. 4) shows that current studies have reported nine antithrombotic regimens for the prevention of PVST-POM 1 after splenectomy in patients with cirrhosis, among which the comparisons between LMWH + warfarin + APT and LMWH + APT were the most frequently studied. The results of the network meta-analysis indicated that LMWH + rivaroxaban and rivaroxaban + APT had the best therapeutic effects, with no significant difference between them. Rivaroxaban + APT was significantly superior to LMWH + warfarin + APT, LMWH + APT, rivaroxaban, APT, and placebo. LMWH + rivaroxaban was more effective than rivaroxaban alone. LMWH + apixaban + APT did not have superior therapeutic effects, as detailed in Table 2. The SUCRA demonstrated that LMWH + rivaroxaban and rivaroxaban + APT might be most effective in preventing PVST-POM 1 after splenectomy (Fig. 5). The comparison-adjusted funnel plot was approximately symmetrical, and the *P-*values of both Egger’s and Begg’s tests were >0.05 (*P* = 0.189, *P* = 0.151), suggesting no publication bias or small sample effects (Supplementary Digital Content Figure S4, available at: http://links.lww.com/JS9/F135).

**Table 2:** Network meta-analysis results for PVST-POM 1 incidence with different antithrombotic regimens

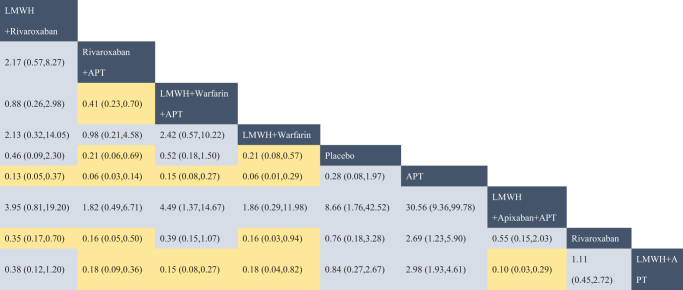

Blue boxes indicated different interventions, and the remaining boxes were the results of pairwise comparisons between different interventions, where statistically significant pairwise comparisons of interventions were highlighted in yellow. Reading from left to right, both RR and its 95% CI were < 1, indicating a statistically significant difference.

#### PVST-POM 3

##### Direct meta-analysis

**Figure 6.**
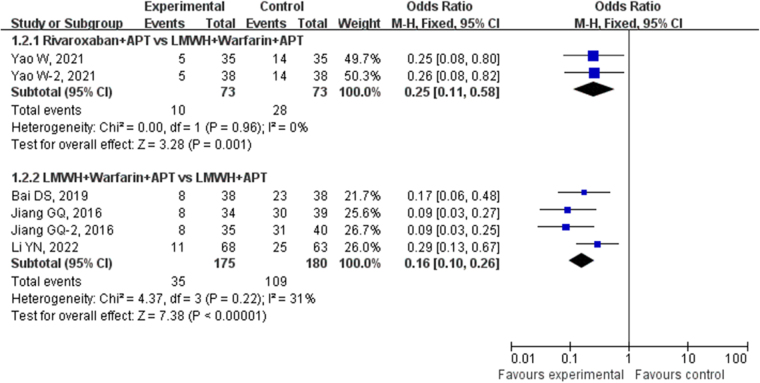
Direct meta-analysis comparison of different antithrombotic regimens in preventing PVST-POM 3.

A total of 2 studies involving 146 patients with cirrhosis and portal hypertension who underwent splenectomy were included in the present analysis. These studies compared the incidence rates of PVST-POM 3 between the use of rivaroxaban + APT and LMWH + warfarin + APT for prevention. No statistical heterogeneity was discovered among the included studies (*P* = 0.96, *I*^2^ = 0%), so a fixed-effects model was used for the analysis. The results of the direct meta-analysis indicated that the incidence of PVST-POM 3 in the rivaroxaban + APT group (13.70%) was significantly lower than that in the LMWH + warfarin + APT group (38.36%), and the difference between the two groups was statistically significant (OR: 0.25, 95% CI: 0.11–0.58, *P* = 0.001), as shown in Figure 6. A total of 4 studies involving 355 patients with cirrhosis and portal hypertension who underwent splenectomy were included in the present analysis. These studies compared the occurrence of PVST-POM 3 between LMWH + APT and LMWH + warfarin + APT for prevention. No statistical heterogeneity was found among the included studies (*P* = 0.22, *I*^2^ = 31%), so a fixed-effects model was used for the analysis. The direct meta-analysis results indicated that the incidence of PVST-POM 3 in the LMWH + warfarin + APT group (20.00%) was significantly lower than that in the LMWH + APT group (60.56%), and the difference between the two groups was statistically significant (OR: 0.16, 95% CI: 0.10–0.26, *P* < 0.00001), as shown in Figure 6.

Sensitivity analysis was performed on the incidence of PVST-POM 3 in the rivaroxaban + APT group vs the LMWH + warfarin + APT group (Supplementary Digital Content Figure S5, available at: http://links.lww.com/JS9/F135) and the LMWH + warfarin + APT group vs the LMWH + APT group (Supplementary Digital Content Figure S6, available at: http://links.lww.com/JS9/F135), and the results revealed no substantial changes in the pooled effect estimate, suggesting that the meta-analysis results were reliable.

##### Network meta-analysis

**Figure 7.**
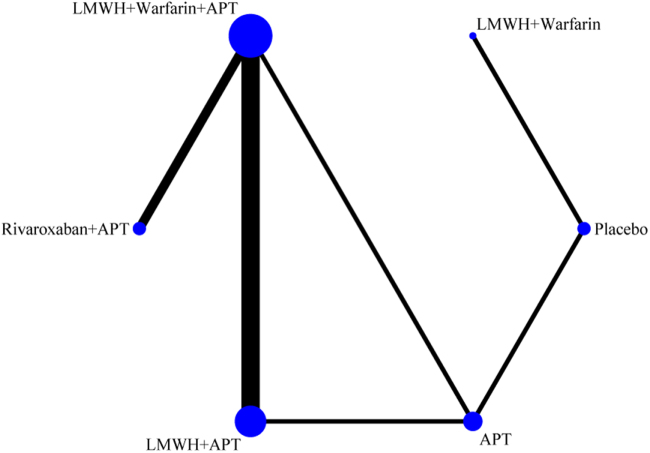
Network diagram of the occurrence of PVST-POM 3 under different antithrombotic regimens.

**Figure 8.**
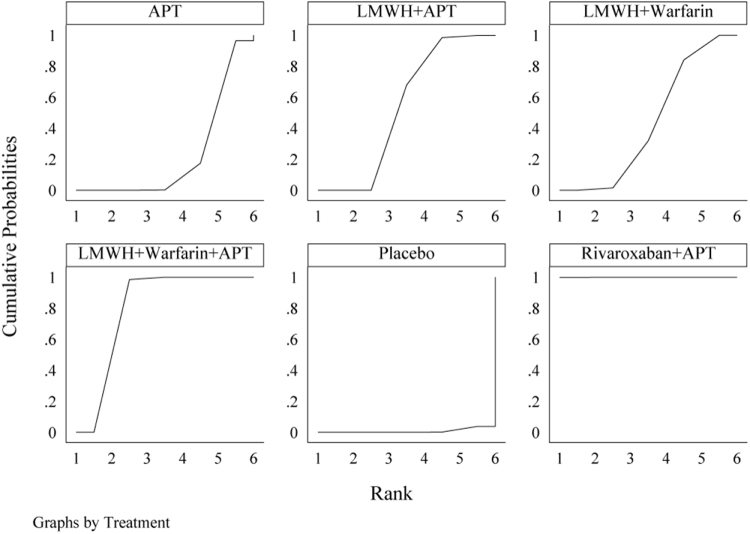
SUCRA of the occurrence of PVST-POM 3 under different antithrombotic regimens.

The network evidence diagram showed that the current studies reported six antithrombotic regimens for the prevention of PVST-POM 3 after splenectomy in cirrhotic patients. The most frequent comparison was between LMWH + warfarin + APT and LMWH + APT (Fig. 7). The results of the network meta-analysis demonstrated that rivaroxaban + APT was significantly more effective than the other regimens in preventing PVST-POM 3. LMWH + warfarin + APT, LMWH + warfarin, and LMWH + APT were significantly more effective than the placebo. LMWH + warfarin + APT was significantly more effective than LMWH + warfarin and LMWH + APT. Additionally, the differences between the remaining treatment regimens were not statistically significant, as detailed in Table 3. The SUCRA showed that rivaroxaban + APT was the most effective regimen for preventing PVST-POM 3 following splenectomy, while single-APT was the least effective, except for the placebo (Fig. 8). The comparison-adjusted funnel plot was approximately symmetrical, and the *P-*values of both Egger’s and Begg’s tests were >0.05 (*P* = 0.164, *P* = 0.128), suggesting that there was no publication bias or small sample effects (Supplementary Digital Content Figure S7, available at: http://links.lww.com/JS9/F135).

**Table 3:** Network meta-analysis results for PVST-POM 3 incidence with different antithrombotic regimens

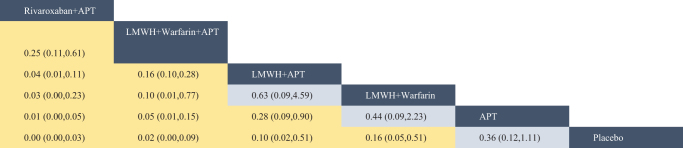

Blue boxes indicated different interventions, and the remaining boxes were the results of pairwise comparisons between different interventions, where statistically significant pairwise comparisons of interventions were highlighted in yellow. Reading from left to right, both RR and its 95% CI were < 1, indicating a statistically significant difference.

#### PVST-POM 6

##### Direct meta-analysis

**Figure 9.**
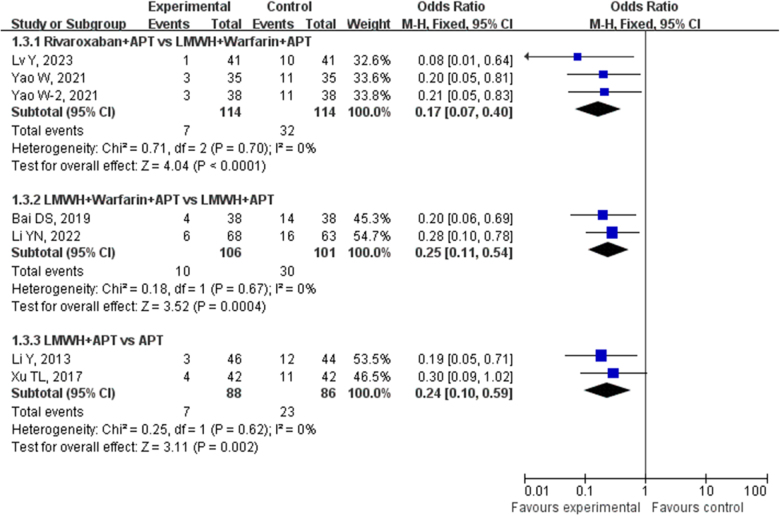
Direct meta-analysis comparison of different antithrombotic regimens in preventing PVST-POM 6.

A total of 3 studies involving 228 patients with cirrhosis and portal hypertension who underwent splenectomy were included in the present analysis. These studies compared the occurrence of PVST-POM-6 between the use of rivaroxaban + APT and LMWH + warfarin + APT for prevention. No statistical heterogeneity was discovered among the included studies (*P* = 0.70, *I*^2^ = 0%), so a fixed-effects model was used for the analysis. The results of the direct meta-analysis indicated that the incidence of PVST-POM 6 in the rivaroxaban + APT group (6.14%) was significantly lower than that in the LMWH + warfarin + APT group (28.07%), and the difference between the two groups was statistically significant (OR: 0.17, 95% CI: 0.07–0.40, *P* = 0.001), as shown in Figure 9. Two studies involving 207 patients with cirrhosis and portal hypertension who underwent splenectomy were included in the current analysis. These studies compared the occurrence of PVST-POM 6 between LMWH + APT and LMWH + warfarin + APT for prevention. No statistical heterogeneity was found among the included studies (*P* = 0.67, *I*^2^ = 0%), so a fixed-effects model was used for the analysis. The direct meta-analysis results revealed that the incidence of PVST-POM-6 in the LMWH + warfarin + APT group (9.43%) was significantly lower than that in the LMWH + APT group (29.70%), and the difference between the two groups was statistically significant (OR: 0.25, 95% CI: 0.11–0.54, *P* = 0.0004), as shown in Figure 9. Two studies involving 174 patients with cirrhosis and portal hypertension who underwent splenectomy were included in the present analysis. These studies compared the occurrence of PVST-POM 6 between LMWH + APT and APT for prevention. No statistical heterogeneity was found among the included studies (*P* = 0.62, *I*^2^ = 0%), so a fixed-effects model was used for the analysis. The direct meta-analysis results revealed that the incidence of PVST-POM-6 in the LMWH + APT group (7.95%) was significantly lower than that in the APT group (26.74%), and the difference between the two groups was statistically significant (OR: 0.24, 95% CI: 0.10–0.59, *P* = 0.002), as shown in Figure 9.

Sensitivity analysis was performed on the incidence of PVST-POM 6 in the rivaroxaban + APT group vs the LMWH + warfarin + APT group (Supplementary Digital Content Figure S8, available at: http://links.lww.com/JS9/F135), the LMWH + warfarin + APT group vs the LMWH + APT group (Supplementary Digital Content Figure S9, available at: http://links.lww.com/JS9/F135), and the LMWH + APT group vs the APT group (Supplementary Digital Content Figure S10, available at: http://links.lww.com/JS9/F135). The results revealed no substantial changes in the pooled effect estimate, suggesting that the meta-analysis results were reliable.

##### Network meta-analysis

**Figure 10.**
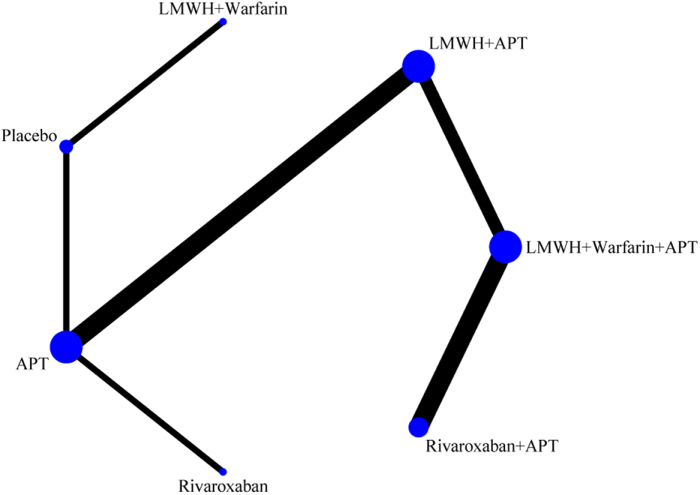
Network diagram of the occurrence of PVST-POM 6 under different antithrombotic regimens.

**Figure 11.**
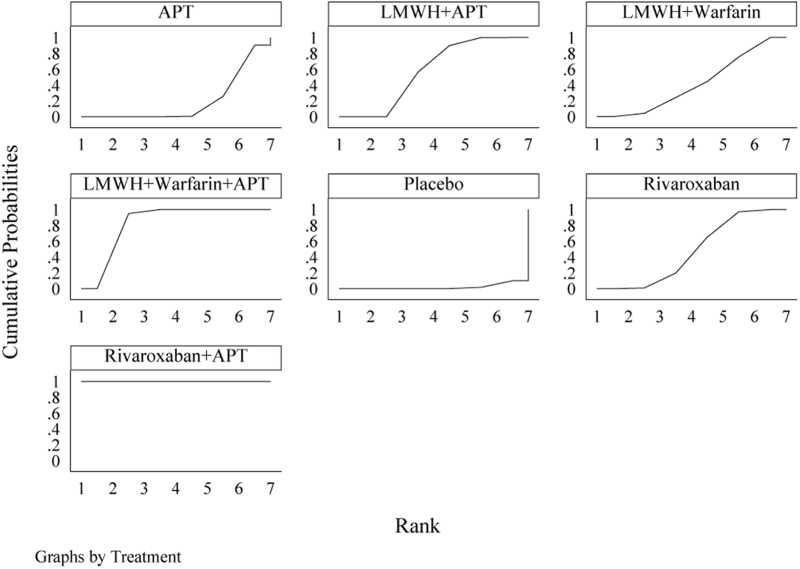
SUCRA of the occurrence of PVST-POM 6 under different antithrombotic regimens.

The network evidence diagram showed that the current studies reported seven antithrombotic regimens for preventing PVST-POM 6 after splenectomy in cirrhotic patients. The most frequent comparison was between LMWH + APT and APT (Fig. 10). The results of the network meta-analysis demonstrated that rivaroxaban + APT was significantly more effective than the other regimens in preventing PVST-POM 6. Rivaroxaban, LMWH + warfarin + APT, LMWH + warfarin, and LMWH + APT were significantly more effective than placebo. Rivaroxaban, LMWH + warfarin + APT, and LMWH + APT were significantly more effective than APT; LMWH + warfarin + APT was significantly more effective than rivaroxaban; and LMWH + warfarin + APT was significantly more effective than LMWH + APT. Additionally, the differences between the remaining treatment regimens were not statistically significant, as detailed in Table 4. The SUCRA showed that rivaroxaban + APT was the most effective regimen for preventing PVST-POM 6 following splenectomy, while single-APT was the least effective, with the exception of placebo (Fig. 11). The comparison-adjusted funnel plot was asymmetric, the *P*-value of Egger’s test (*P* = 0.039) was < 0.05, and the *P*-value of Begg’s test (*P* = 0.060) was >0.05, suggesting potential publication bias or a small sample effect (Supplementary Digital Content Figure S11, available at: http://links.lww.com/JS9/F135).

**Table 4:** Network meta-analysis results for PVST-POM 6 incidence with different antithrombotic regimens

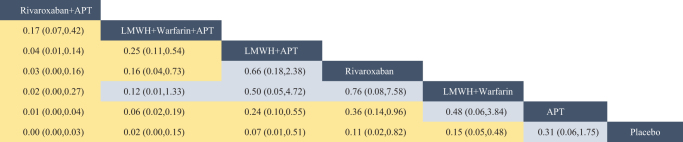

Blue boxes indicated different interventions, and the remaining boxes were the results of pairwise comparisons between different interventions, where statistically significant pairwise comparisons of interventions were highlighted in yellow. Reading from left to right, both RR and its 95% CI were < 1, indicating a statistically significant difference.

#### Incidence of bleeding

##### Direct meta-analysis

**Figure 12.**
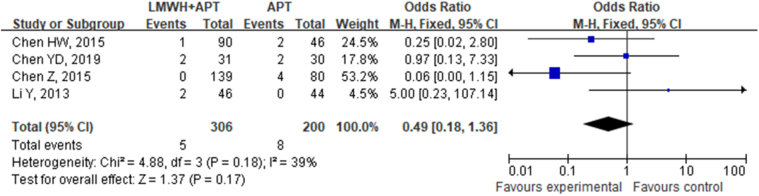
Direct meta-analysis comparison of different antithrombotic regimens in bleeding incidence.

A total of 4 studies involving 506 patients with cirrhosis and portal hypertension who underwent splenectomy were included in the present analysis. These studies compared the occurrence of bleeding between the use of LMWH + APT and APT. No statistical heterogeneity was found among the included studies (*P* = 0.18, *I*^2^ = 39%), so a fixed-effects model was used for the analysis. The direct meta-analysis results revealed a similar incidence of bleeding in the LMWH + APT group (1.63%) to the APT group (4.00%), with a difference between the two groups that was not statistically significant (OR: 0.49, 95% CI: 0.18–1.36, *P* = 0.17), as shown in Figure 12.

Sensitivity analysis of the incidence of bleeding in the LMWH + APT group vs the APT group was performed (Supplementary Digital Content Figure S12, available at: http://links.lww.com/JS9/F135), and the results revealed no substantial changes in the pooled effect estimate, suggesting that the meta-analysis results were reliable.

##### Network meta-analysis

**Figure 13.**
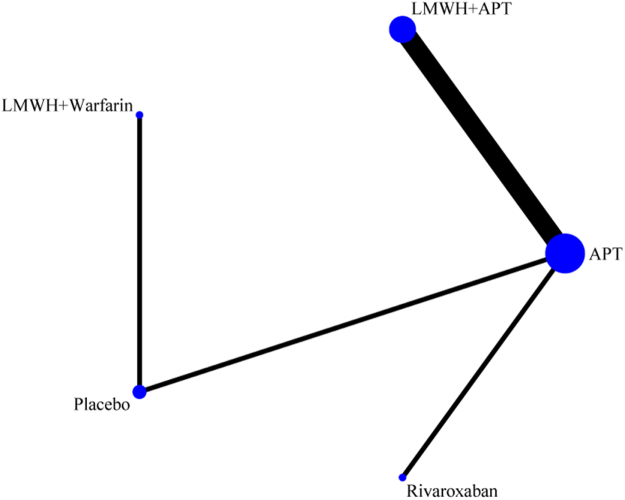
Network diagram of the incidence rate of bleeding under different antithrombotic regimens.

**Figure 14.**
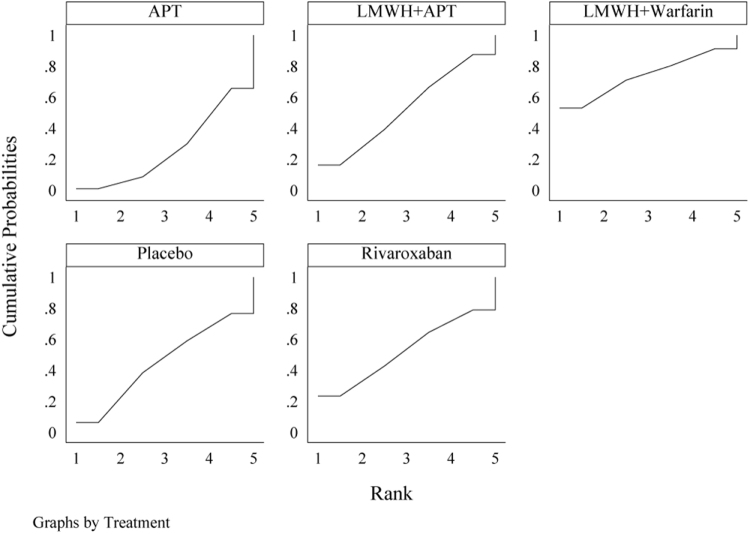
SUCRA of the incidence rate of bleeding under different antithrombotic regimens.

The network evidence diagram shows that the current studies reported five antithrombotic regimens for preventing PVST in cirrhotic patients following splenectomy, with a focus on bleeding incidence. The most frequent comparison was between LMWH + APT and APT (Fig. 13). The network meta-analysis results revealed no statistically significant differences in the incidence of bleeding between the various treatment regimens, as detailed in Table 5. The SUCRA analysis revealed that LMWH + warfarin was associated with the lowest probability of bleeding (Fig. 14). The comparison-adjusted funnel plot was approximately symmetrical, and the *P*-values of both Egger’s and Begg’s tests were >0.05 (*P* = 0.724, *P* = 0.453), suggesting that there was no publication bias or small sample effects (Supplementary Digital Content Figure S13, available at: http://links.lww.com/JS9/F135).

**Table 5:** Network meta-analysis results for the incidence rate of bleeding with different antithrombotic regimens

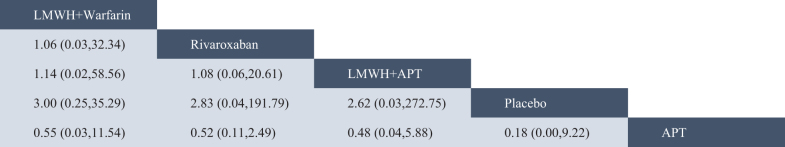

Blue boxes indicated different interventions, and the remaining boxes were the results of pairwise comparisons between different interventions, where statistically significant pairwise comparisons of interventions were highlighted in yellow. Reading from left to right, both RR and its 95% CI were < 1, indicating a statistically significant difference.

#### Course of antithrombotic prophylaxis

##### Meta-analysis

**Figure 15.**
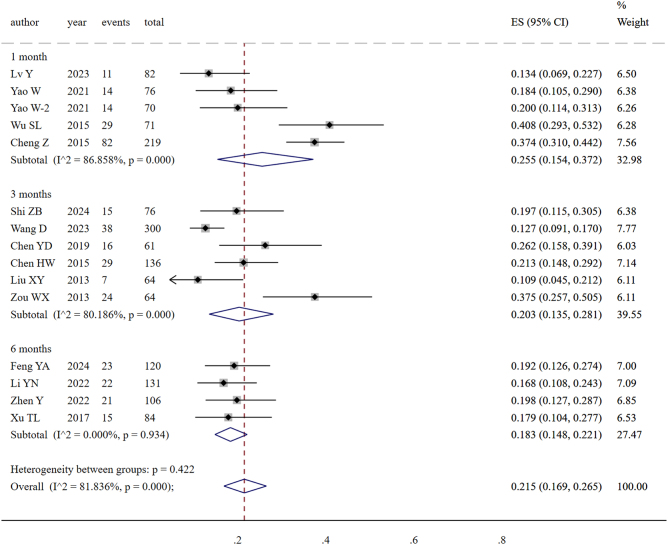
Comparison of the incidence of PVST at the last follow-up after different courses of postoperative prophylactic antithrombotic therapy.

A total of 15 studies included 1361 patients with liver cirrhosis and portal hypertension who underwent splenectomy. These patients received postoperative prophylactic antithrombotic therapy for 1 month (219 patients), 3 months (701 patients), or 6 months (441 patients). The included studies showed high heterogeneity (*P* = 0.000, *I*^2^ = 81.84%), so a random effects model was used. The results of the meta-analysis indicated that the incidence of postoperative PVST in patients with liver cirrhosis and portal hypertension who underwent splenectomy was 21.5% (95% CI: 16.9–26.5%), as shown in Figure 15. The funnel plot suggested no publication bias, and the results of Egger’s test (*P* = 0.716) also indicated the absence of publication bias, as shown in Supplementary Digital Content Figure S14, available at: http://links.lww.com/JS9/F135.

Data analysis revealed that the incidence of PVST at the last follow-up was 28.96% (82/219) in patients with a 1-month prophylactic antithrombotic course, 18.40% (129/701) in those with a 3-month course, and 18.36% (81/441) in those with a 6-month course. Therefore, 3 months of postoperative prophylactic antithrombotic therapy for patients with liver cirrhosis and portal hypertension undergoing splenectomy could significantly reduce the incidence of PVST, whereas extending the prophylactic antithrombotic course to 6 months provided no further reduction of the incidence of PVST.

## Discussion

This meta-analysis included 19 studies with a total of 1968 patients. The results showed that prophylactic antithrombotic therapy was able to significantly reduce the incidence of PVST after splenectomy in patients with cirrhosis. According to the follow-up data at POM 1, POM 3, and POM 6, overall, the efficacy of different regimens in preventing PVST ranked as anticoagulation combined with APT > anticoagulation therapy alone > APT alone > placebo. The network meta-analysis results revealed no significant differences in bleeding risk among the different treatment regimens.

Previous meta-analyses have demonstrated that prophylactic anticoagulation can significantly reduce the occurrence of postoperative PVST, and early intervention is superior to delayed intervention^[39,40]^. In this study, among patients without pharmacological intervention, the incidence of PVST within 6 months after surgery was approximately 27.5–43.47%, with the peak period being the first 3 months after surgery. Prophylactic anticoagulation was initiated within 12–72 hours after surgery, whereas APT was generally initiated when the platelet count exceeded 300 or 500 × 10^9^/L. Direct meta-analysis of the efficacy results at POM 1 revealed that the preventive effect of anticoagulation + APT was generally good, among which rivaroxaban + APT provided the best effect. The SUCRA values indicated that LMWH + rivaroxaban and rivaroxaban + APT were the best options, and there was no significant difference between them. The preventive effect of the rivaroxaban + APT group significantly differed from that of the LMWH + warfarin + APT, LMWH + APT, rivaroxaban, and APT groups. The efficacy results at POM 3 and POM 6 were consistent, with the anticoagulation combined with APT group showing superior preventive efficacy compared with the other groups. The SUCRA plot indicated that rivaroxaban + APT had the best preventive effect. This finding is consistent with the results of the network meta-analysis in the study reported by Yuan-ming Xing *et al*^[41]^. Currently, the most common clinical prophylactic regimen is LMWH followed by warfarin combined with APT. However, monitoring the use of warfarin is challenging in cirrhotic patients with elevated international normalized ratio (INR) levels^[42]^, and frequent monitoring can affect patients’ compliance with medication. Antithrombin III activity gradually decreases to less than 60% in Child‒Pugh class B/C patients^[43]^, affecting the application of LMWH. In real-world practice, the prescription of DOACs, mainly rivaroxaban, for prophylaxis has gradually increased because of their safety, efficacy, and ease of use^[44]^. We are the first group to compare the postoperative PVST prevention effects of rivaroxaban combined with APT. Compared with the traditional LMWH sequential warfarin combined with the APT regimen, the rivaroxaban + APT group had a PVST incidence of 28.95% at POM 1 (OR 0.41, CI 0.23–0.70, *P* = 0.001), 13.70% at POM 3 (OR 0.25, CI 0.11–0.58, *P* = 0.001), and 6.14% at POM 6 (OR 0.17, CI 0.07–0.40, *P* = 0.001), indicating significantly greater prevention efficacy compared with the other groups. APT alone provided the worst preventative effect. Currently, the 2021 EHRA guidelines recommend the use of rivaroxaban only in Child‒Pugh class A patients and not in class B/C patients^[45]^. From a pharmacological perspective, approximately 65% of rivaroxaban is metabolized by the hepatic enzymes CYP3A4 and CYP2J2. As the severity of cirrhosis increases and liver function decreases, rivaroxaban exposure increases, theoretically increasing the risk of bleeding. However, with the increase of clinical evidence, the 2024 ISTH recommended that patients with Child–Pugh grade B could also be treated at the standard dose^[44]^. However, among cirrhotic patients with other indications for anticoagulation, the efficacy of rivaroxaban in preventing thromboembolic events has been found to be consistent with that of warfarin, with no observed increase in bleeding risk^[46,47]^. Among the literature included in the present analysis, the apixaban + APT group also demonstrated effectiveness in the prevention of PVST. However, owing to its small sample size, no significant difference was observed in the results. Additional clinical trials are still needed to verify these findings.

Drugs for the prevention of PVST include anticoagulants and antiplatelet agents. The dosages of DOACs among anticoagulants are basically consistent with those used for VTE prevention, mainly 10 mg qd for rivaroxaban and 2.5 mg bid for apixaban, and warfarin is mostly administered at a dosage that maintains the INR at 2–3. LMWH has both prophylactic and therapeutic dosages for the treatment of VTE. Antiplatelet agents are initiated when the platelet count is >300 or 500 × 10^9^/L. The dosage range of aspirin is 75–100 mg qd, and dipyridamole is mostly given at 25 mg tid. Owing to the wide variety of antithrombotic regimens and dosages, statistical analysis is not feasible, so the above dosages can be referred to for selection. There is currently no consensus on the duration of thromboprophylaxis for PVST. The prophylactic durations in the included studies were 1, 3, 6, and 12 months. In patients without drug intervention, the incidence of PVST reached as high as approximately 40% within 3 months and then gradually decreased. In the present study, the incidences of PVST at the last follow-up for patients receiving antithrombotic therapy for 1, 3, and 6 months were 28.96%, 18.4%, and 18.36%, respectively. These findings suggested that 3 months of prophylactic antithrombotic therapy after splenectomy for cirrhotic portal hypertension could significantly reduce the incidence of PVST, whereas extending the prophylactic antithrombotic course to 6 months provided no further reduction of the incidence of PVST.

Patients with cirrhosis often experience bleeding due to esophagogastric varices and have an increased risk of bleeding due to impaired synthesis of coagulation factors and platelets. However, current studies on the prevention^[48]^ and treatment^[49,50]^ of PVST in patients with cirrhosis have shown that VK1, LMWH, and DOACs do not increase the risk of bleeding in these patients. In the IMPORTAL study^[51]^, among patients with cirrhosis and PVST, the global incidence of bleeding events was similar between those who received anticoagulation therapy and those who did not (19.0% vs 15.6%). Although the incidence of portal hypertension-related bleeding was similar between the two groups, the incidence of nonportal hypertension-related bleeding, mainly gastrointestinal bleeding, was greater in the anticoagulation group. This phenomenon is related to hemostatic disorders in cirrhotic patients, where esophagogastric varices, gastrointestinal ulcers, and other conditions increase the likelihood and severity of gastrointestinal bleeding. Similarly, a meta-analysis of bleeding risk in cirrhotic patients receiving DOACs revealed that DOACs are associated with a lower risk of bleeding, with incidence rates of any bleeding, major bleeding, fatal bleeding, gastrointestinal bleeding, and intracranial bleeding of 13%, 6%, 0%, 8%, and 0%, respectively^[52]^. Patients with longer treatment cycles (>180 days) and those classified as Child‒Pugh class C had a higher incidence of bleeding. However, there are still reports that the incidence of bleeding appears to increase in patients with longer anticoagulant treatment durations (>180 days) and higher Child‒Pugh classifications^[53]^. Patients with such a high risk of bleeding should be managed in two ways: patient factors and drug treatment factors. Based on current evidence, we have provided recommendations for the treatment regimen and course of PVST prophylaxis. Patients should also undergo regular endoscopic evaluation to correct risk factors that easily lead to bleeding in a timely manner. When necessary, non-β-blockers or endoscopic treatment should be combined for the primary prevention of GEV bleeding^[54]^.

This study has several strengths. First, to our knowledge, this is the first study to include nine different clinically commonly used antithrombotic regimens, including DOACs such as rivaroxaban and apixaban, and to quantitatively compare their efficacy and safety in preventing PVST in patients with cirrhosis and portal hypertension undergoing splenectomy, thereby addressing gaps present in the existing published studies. Second, we analyzed four clinical outcome indicators, particularly the incidence of bleeding, and comprehensively evaluated the advantages and disadvantages of different antithrombotic regimens from multiple perspectives and dimensions, providing evidence-based medical support for clinicians to select individualized antithrombotic regimens according to the different clinical characteristics of patients. Third, we explored the effects of different durations of prophylactic antithrombotic therapy on the occurrence of PVST after splenectomy in patients with liver cirrhosis and portal hypertension. Moreover, we conducted an extensive literature search and included a larger number and more recent studies compared with other research. Finally, the prevention of PVST in patients with cirrhosis and portal hypertension undergoing splenectomy is critically important and urgently needed in clinical practice, yet few meta-analyses have addressed this issue, especially regarding the efficacy and safety of DOACs, highlighting the special significance of this study.

This study also has certain limitations. (1) This study included retrospective studies, which may have affected the homogeneity, similarity, and consistency required for network meta-analysis. (2) Due to limitations in the included studies, it was not possible to compare the efficacy and safety of some antithrombotic regimens for certain outcome indicators, such as the efficacy of rivaroxaban in PVST-POM 3 and the bleeding incidence with rivaroxaban + APT. (3) Some of the included studies had small sample sizes and short follow-up durations, which may have led to an insufficient assessment of the impact on clinical outcomes. (4) Most of the included studies were single-center studies, which may introduce certain biases into the results. (5) For the clinical outcome of PVST-POM 6, publication bias was identified in the included articles, which may have had a certain impact on the research results. (6) The dosage had a significant impact on the formation of postoperative PVST in patients with liver cirrhosis and portal hypertension who underwent splenectomy. However, owing to the excessive classification of drug dosages in the included studies, making it impossible to categorize them, this study did not further analyze the influence of drug dosages. (7) The studies included in our research were all from China, which may introduce bias to the results.

## Conclusion

In conclusion, for patients with cirrhosis and portal hypertension undergoing splenectomy and with Child‒Pugh class A‒B liver function, prophylactic antithrombotic regimens combining anticoagulation and APT, especially rivaroxaban + APT, demonstrated the best efficacy, which could significantly reduce the incidence of PVST and not increase the incidence of bleeding. Prophylactic antithrombotic regimens using APT alone had effects similar to those of placebo; both were less effective and significantly increased the incidence of PVST in this patient population. A 3-month postoperative prophylactic antithrombotic therapy may be the optimal preventive course. Clinicians should select individualized antithrombotic regimens based on the specific conditions of each patient in clinical practice. We look forward to prospective, large-scale RCTs to further determine the efficacy and safety of different antithrombotic regimens, providing evidence to optimize the best prevention strategies for PVST in patients with cirrhosis and portal hypertension undergoing splenectomy.

## Data Availability

The majority of the data used and generated in this study are available in the manuscript. Requests for any remaining data or analysis codes can be made to the corresponding author upon reasonable request.

## Research registration unique identifying number (UIN)

This study had been registered on the PROSPERO database with the registration number CRD42024627765.
